# Special feature: “current status and future development of organ preservation technology” novel techniques for assessing pulmonary function in cellular ex vivo lung perfusion: a republication of the review published in Japanese Journal of Artificial Organs

**DOI:** 10.1007/s10047-026-01557-8

**Published:** 2026-05-08

**Authors:** Ryo Kosaka, Daisuke Sakota, Hiromichi Niikawa, Yoshinori Okada, Tetsuhito Kigata, Katsuhiro Ohuchi, Eiki Nagaoka, Tomoyuki Fujita, Ichiro Sakanoue, Toshihiro Okamoto

**Affiliations:** 1 Health and Medical Research Institute, National Institute of Advanced Industrial Science and Technology (AIST), 1-2-1 Namiki, Tsukuba, 305-8564 Ibaraki Japan; 2 Thoracic Surgery, Tohoku Medical and Pharmaceutical University, Sendai, Miyagi Japan; 3 Department of Thoracic Surgery, Institute of Development, Aging and Cancer, Tohoku University, Sendai, Miyagi Japan; 4 Laboratory of Veterinary Anatomy, Tokyo University of Agriculture and Technology, Fuchu, Tokyo Japan; 5 Department of Clinical Engineering, Faculty of Medical Science, Juntendo University, Urayasu, Chiba Japan; 6 Department of Cardiovascular Surgery, Institute of Science Tokyo, Bunkyo-ku, Tokyo Japan; 7 Department of Thoracic Surgery, Kyoto University, Kyoto, Kyoto Japan; 8 Department of Thoracic and Cardiovascular Surgery, Cleveland Clinic, Cleveland, OH USA; 9 Department of Inflammation and Immunology, Lerner Research Institute, Cleveland Clinic, Cleveland, OH USA; 10 Transplant Center, Cleveland Clinic, Cleveland, OH USA

**Keywords:** Ex vivo lung perfusion, Pulmonary function assessment, Lung thermography, Optical oxygen saturation imaging, Real-time lung weight measurement

## Abstract

Lung transplantation is the definitive therapy for end-stage respiratory diseases. To expand the donor lung pool, ex vivo lung perfusion (EVLP) has been developed for the assessment of marginal donor lungs. However, current evaluation methods remain limited. This study aimed to develop non-invasive imaging and monitoring techniques for the quantitative and early assessment of pulmonary function during EVLP. Three novel approaches were established: (1) lung thermography during the initial reperfusion period to assess pulmonary function, (2) optical oxygen saturation (SaO₂) imaging to assess pulmonary oxygenation, and (3) real-time lung weight measurement as an early indicator of transplant suitability. Lung thermography revealed that lung surface temperature at 8 min after shunt closure was significantly lower in non-suitable cases than in suitable cases (25.1 ± 0.6 °C vs. 27.8 ± 1.2 °C, *P* < 0.01). Optical SaO₂ imaging demonstrated a strong correlation between lower lobe SaO₂ calculated from SaO₂ imaging and PaO_2_/FiO_2_ (P/F) ratio in the lower pulmonary vein (*R* = 0.855, *P* < 0.01), with SaO₂ being significantly lower in non-suitable cases. Real-time lung weight measurement showed that lung weight gain increased significantly after 40 min in non-suitable cases compared with suitable cases (51.6 ± 46.0 g vs. −8.8 ± 25.7 g, *P* < 0.01). These three approaches proved effective for the quantitative and early assessment of pulmonary function during EVLP. This review was created based on a translation of the Japanese review written in the Japanese Journal of Artificial Organs in 2024 (Vol. 53, No. 3, pp. 216–220).

## Introduction

Lung transplantation has been established as the definitive therapy for patients with end-stage respiratory diseases. However, in Japan, only 119 lung transplants from brain-dead donors were performed in 2023, whereas 572 patients remained on the waiting list, indicating a persistent shortage of donor lungs [1]. In the United States, 3016 lung transplants were performed in 2023. Nevertheless, because of limitations in cold preservation time and donor lung dysfunction during intensive care management, only 18% of donor lungs are clinically utilized for transplantation [2]. To address this issue, ex vivo lung perfusion (EVLP) has been developed and clinically applied, mainly in Europe and North America, for the evaluation of transplant suitability of donor lungs prior to transplantation [3, 4]. However, current evaluation methods during EVLP remain insufficient, which may lead to poor clinical outcomes. Therefore, a quantitative and early functional assessment technique for donor lungs is required to objectively evaluate pulmonary function and expand the donor pool.

This paper describes three novel techniques for the quantitative and early functional assessment of donor lungs during EVLP developed through Japan–U.S. medical-engineering collaboration: (1) lung thermography during the initial reperfusion period to assess pulmonary function, (2) optical oxygen saturation (SaO_2_) imaging for the assessment of pulmonary oxygenation, and (3) real-time lung weight measurement as an early indicator of transplant suitability.

### Developed EVLP system and perfusion conditions

**Fig. 1:**
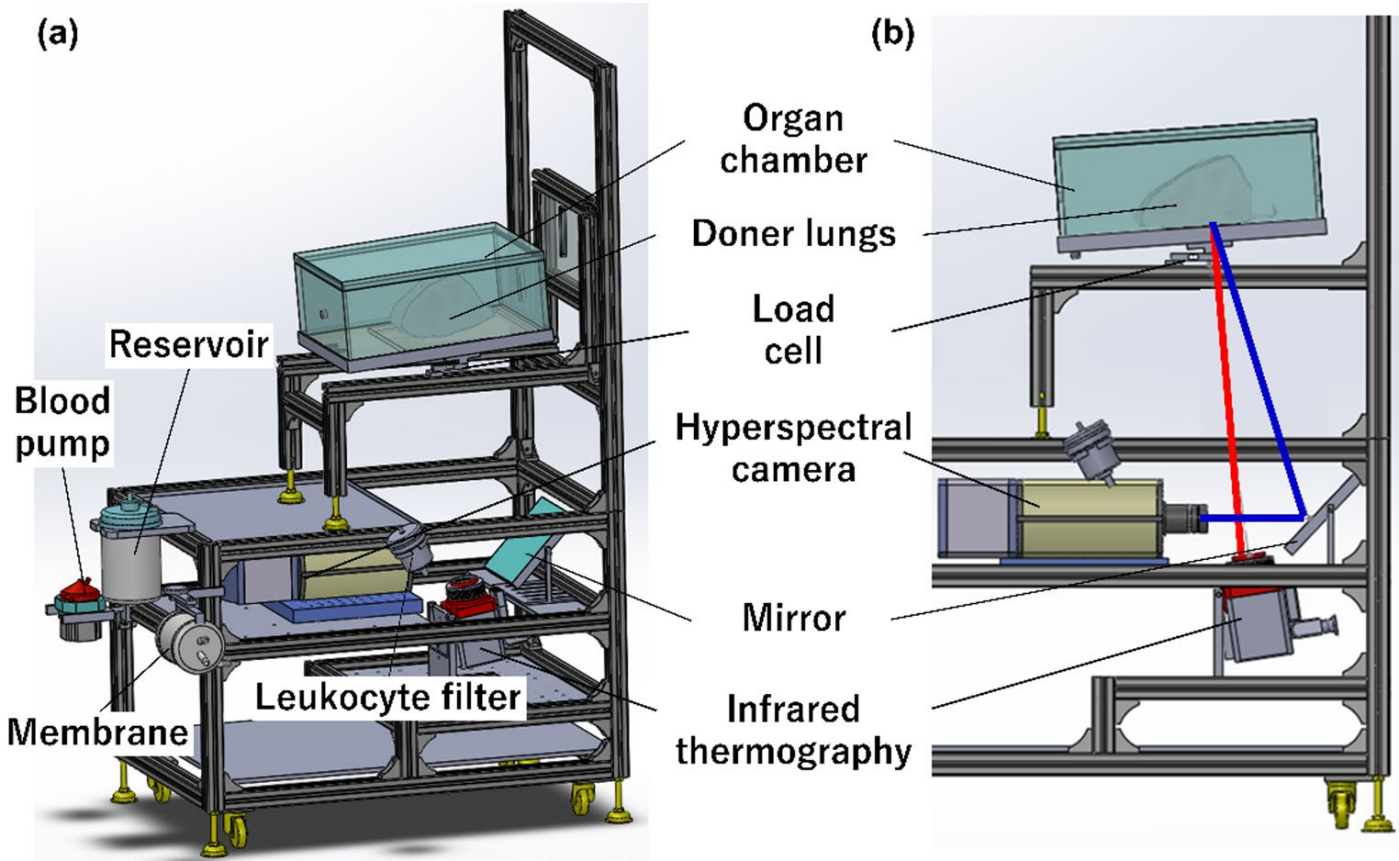
The developed EVLP system

We constructed an EVLP system to evaluate pulmonary function, as shown in Fig. 1. The system consisted of an organ chamber for mounting the donor lungs, a reservoir, a centrifugal blood pump, a membrane oxygenator, a heat exchanger, and a leukocyte filter as components of the EVLP circuit (Fig. 1a). An infrared thermography and a hyperspectral camera were positioned on the bottom side of the organ chamber to evaluate the dorsal side of the lower lobes, where reperfusion injury might occur according to the “gravity model” by West et al. [5] Additionally, load cells were installed at the base of the organ chamber to enable real-time lung weight measurement during EVLP (Fig. 1b).

Fifteen pigs (42–49 kg) were randomly divided into three groups: a control group, and donation after circulatory death (DCD) groups with 60–90 min of warm ischemia (*n* = 5, each). In the control group, the lungs were subjected to 1 h of cold ischemia followed by 2 h of EVLP. In the DCD groups, the lungs underwent 60–90 min of warm ischemia followed by 5 h of cold ischemia prior to EVLP. Some cases overlapped among assessment methods, while others differed between methods.

**Fig. 2:**
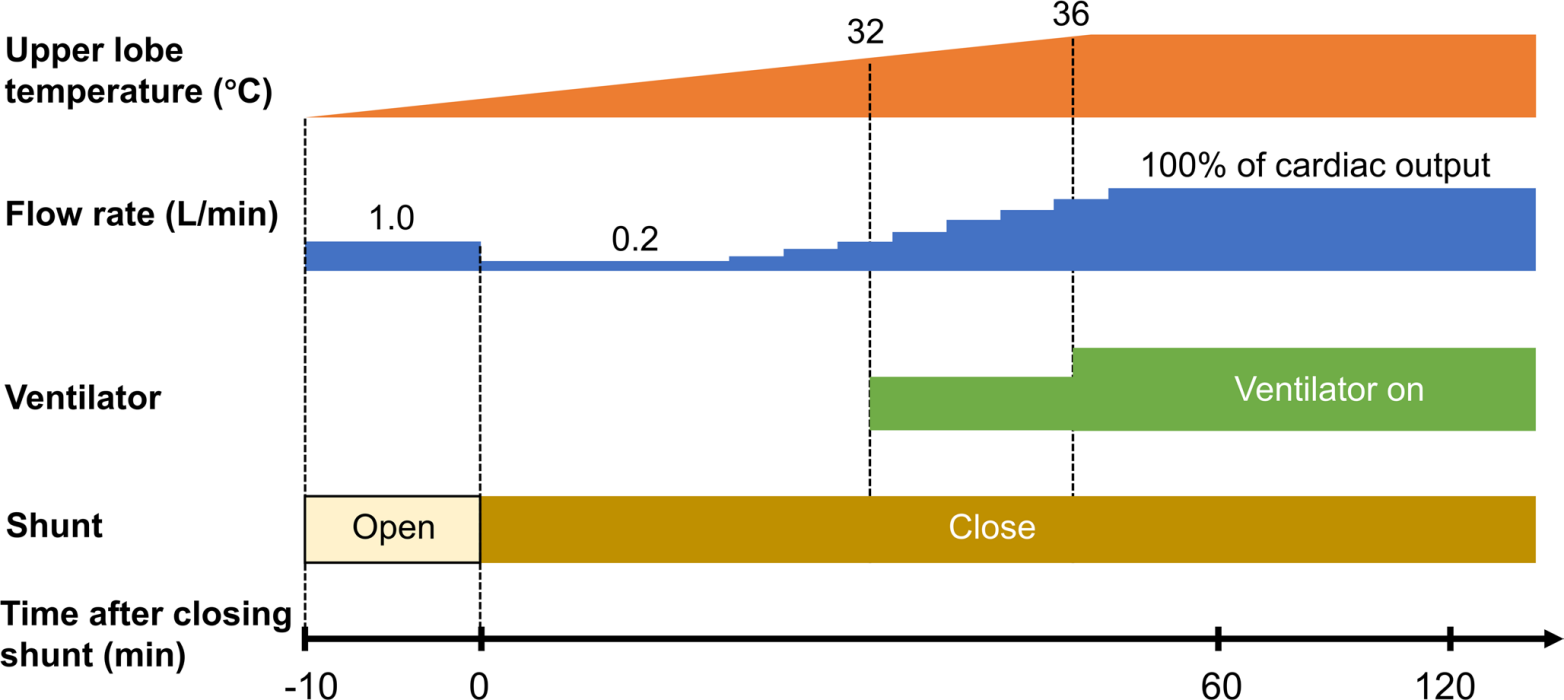
Protocol of Lund-type ex vivo lung perfusion. Perfusion started at a flow rate of 1.0 L/min for 10 min. The flow rate was reduced to 0.2 L/min. Lungs were gradually rewarmed by increasing the flow rate in a stepwise manner until the flow rate reached the estimated cardiac output (70 mL/min/kg). When the upper lobe temperature reached 32 °C, ventilation was started. At 1 and 2 h, blood gas analysis was performed

The lungs were perfused according to the Lund protocol as shown in Fig. 2 [6, 7]. The perfusate consisted mainly of 2.0 L of STEEN solution with 500 mL of packed red blood cells. A pulmonary artery cannula was connected to the circuit, and the left atrium was left open. At the start of perfusion (0 min), the flow rate was maintained at 1.0 L/min, with a shunt between the circuit and the organ chamber opened to prevent sudden reperfusion of the lungs. At 10 min after the initiation of perfusion, the flow rate was reduced to 0.2 L/min, and the shunt was closed. In the evaluation of pulmonary function using lung thermography, the time point when the shunt was closed was defined as time zero. Subsequently, the flow rate was gradually increased in a stepwise manner until the flow rate reached the estimated cardiac output (70 mL/min/kg). Concurrently, the lung was gradually rewarmed to 37 °C using a thermostatic bath connected to a heat exchanger integrated with the membrane oxygenator. When the temperature of the upper lobe reached 32 °C, mechanical ventilation was initiated. Blood gas analyses were performed at 1 h and 2 h after the start of EVLP under different fractions of inspired oxygen (FiO_2_).

After 2 h of EVLP, transplant suitability of the lungs was comprehensively evaluated according to the previously published criteria described by Wierup et al. [8] Lungs were considered non-suitable for transplantation when the PaO_2_/FiO_2_ (P/F) of the left atrium was < 300 mm Hg and when there was a significant deterioration in airway parameters (peak inspiratory pressure, plateau pressure, dynamic compliance and static compliance) and vascular parameters (e.g., flow < 100% of estimated cardiac output). Lungs were also considered non-suitable when significant airway fluid or abnormal visual findings, such as lung edema or hematoma, were detected at the end of perfusion [9–11]. In the present study, all five cases in the control group were judged as suitable for transplantation. In the DCD group with 90 min of warm ischemia, all five cases were determined to be non-suitable, whereas in the DCD group with 60 min of warm ischemia, four cases were suitable, and one case was non-suitable for transplantation.

### Lung thermography during the initial reperfusion period to assess pulmonary function [12]

To evaluate impaired pulmonary circulation caused by reperfusion injury during the initial perfusion phase, the lung surface temperature was continuously recorded using an infrared thermography (R550Pro, Nippon Avionics Co., Ltd., Kanagawa, Japan). The thermography camera had a thermal resolution of 640 × 480 pixels, a sampling frequency of 30 Hz, and a sensitivity of 0.025 °C. The lung surface temperature on the dorsal surface of the lower lobes, where pulmonary edema tends to occur, was monitored through a special transparent resin cover (GAT, Asahi Kasei Advance Co., Ltd., Tokyo, Japan) installed at the bottom of the organ chamber, because standard acrylic resin does not transmit infrared radiation [13].

**Fig. 3:**
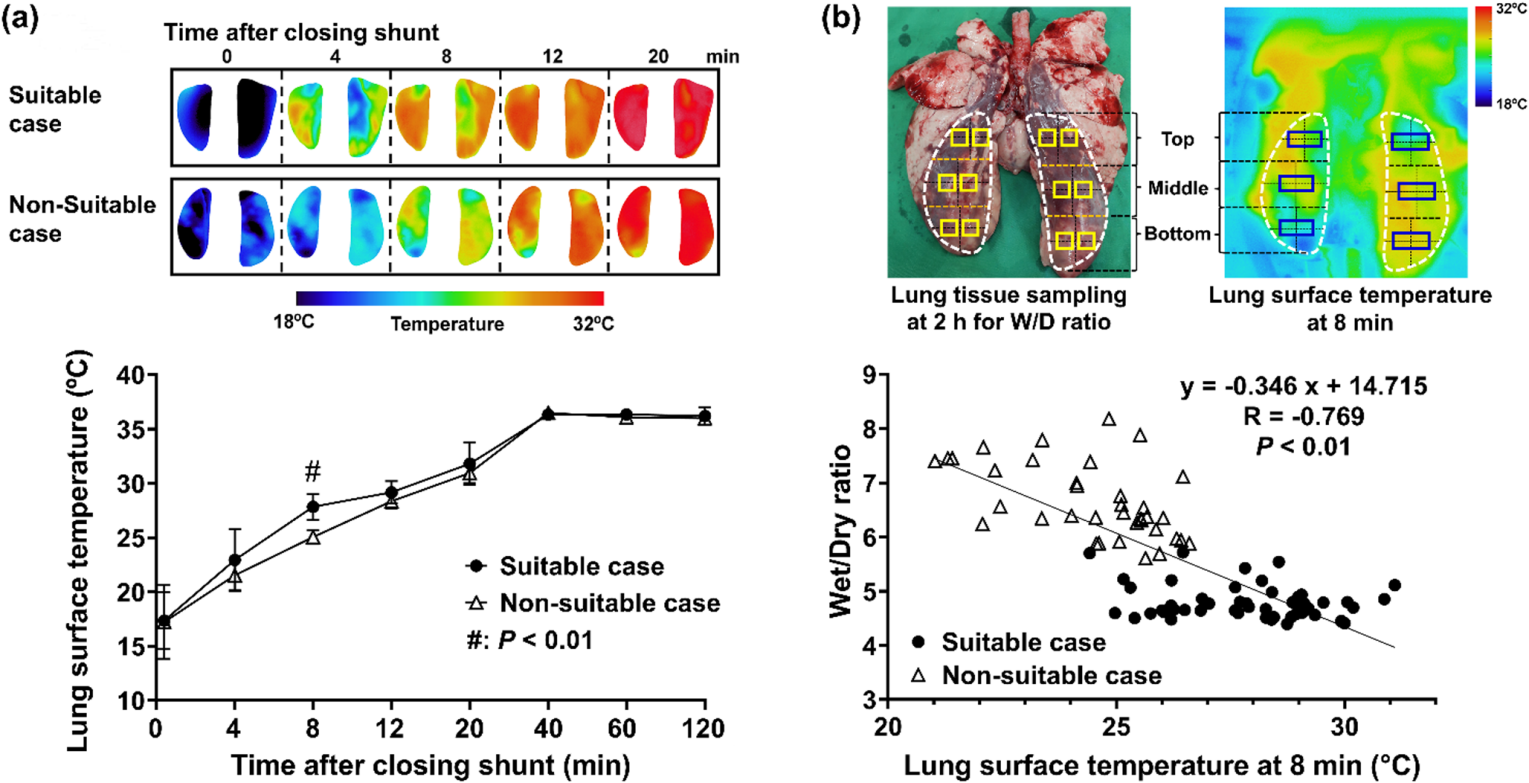
Lung surface temperature measured by infrared thermography for the assessment of pulmonary function during EVLP. **a** Lung surface temperature in suitable and non-suitable cases. **b** Correlation between the lung surface temperature at 8 min and the W/D ratio in the lower lobes after 2 h of EVLP

Figure 3a shows the typical time-dependent changes in temperature distribution and average lung surface temperature measured by infrared thermography in suitable and non-suitable cases. From 0 to 8 min after shunt closure, the lung surface temperature of the lower lobes increased uniformly in suitable cases, whereas the peripheral regions of the lower lobes showed a restricted temperature increase in non-suitable cases. At 8 min, the average lung surface temperature in non-suitable cases was significantly lower than that in suitable cases (25.1 ± 0.6 °C vs. 27.8 ± 1.2 °C, *P* < 0.01). The cut-off value of lung surface temperature at 8 min for determining suitability for transplant was 26 °C, and the area under the receiver operating characteristic (ROC) curve (AUC) was 1.0. From 12 to 120 min, there was no significant difference in lung surface temperature between the two types of cases. As shown in Fig. 3b, a significant negative correlation was observed between lung surface temperature at 8 min and the wet/dry (W/D) ratio in the upper, middle, and lower regions of both lower lobes (*R* = − 0.769, *P* < 0.01). These results indicate that regions with the lower surface temperature at 8 min result in advanced pulmonary edema at the end of EVLP.

These findings suggest that lung surface temperature at 8 min after shunt closure measured by infrared thermography can serve as an early and quantitative indicator of impaired pulmonary circulation during EVLP.

### Optical oxygen saturation imaging for assessment of pulmonary oxygenation [14]

To quantitatively evaluate the regional gas-exchange potential of the lower lobes, optical SaO_2_ imaging during EVLP was performed using a hyperspectral camera (HSi-300, Gooch & Housego Inc., Ilminster, UK). The hyperspectral camera is equipped with an integrated spectrophotometer that acquires wavelength-resolved spectral data for each pixel in a two-dimensional image. SaO_2_ imaging of the lower lobes was determined by analyzing the absorption spectra of hemoglobin obtained from the multispectral images of the lower lobes.

**Fig. 4:**
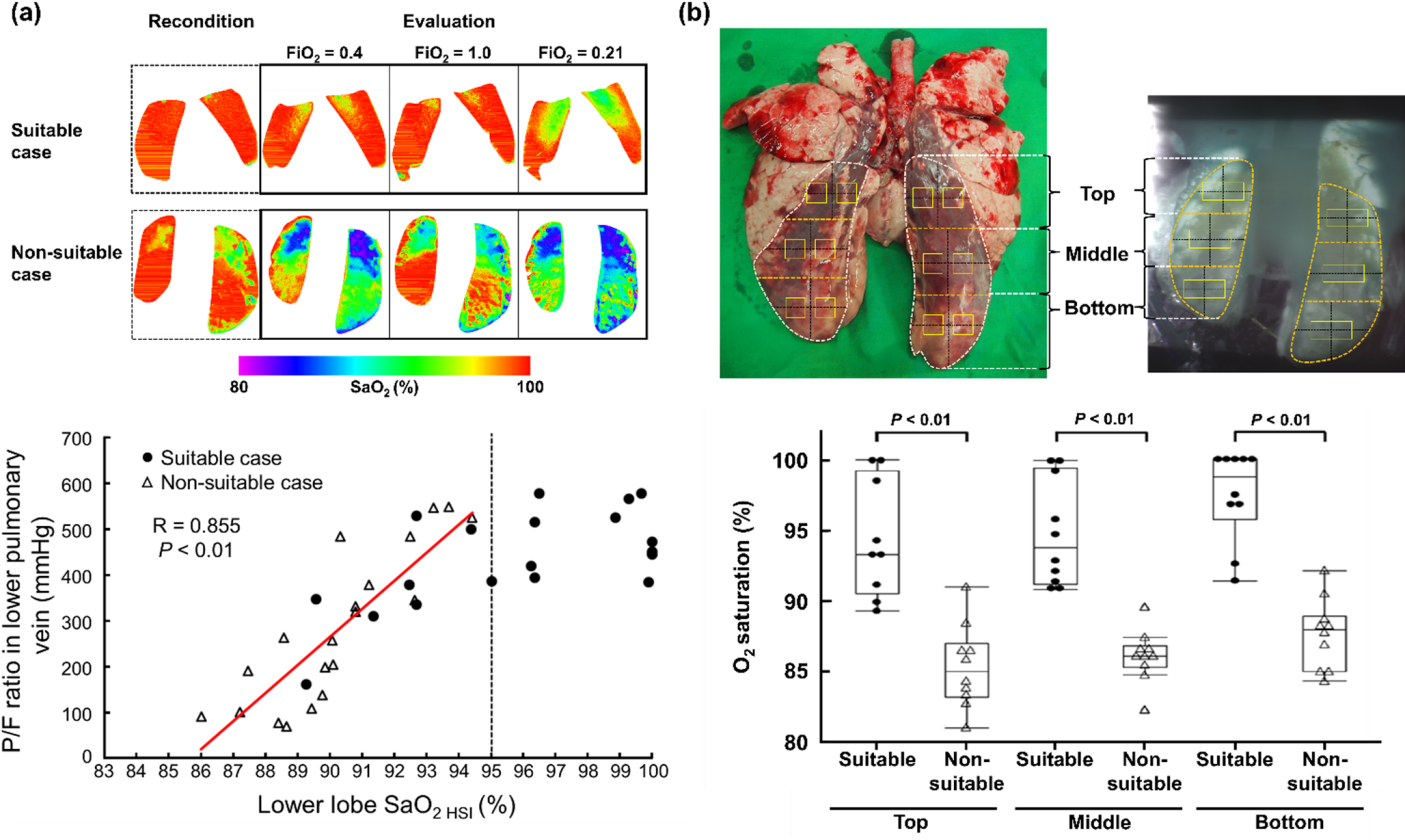
Optical oxygen saturation imaging for the assessment of pulmonary oxygenation. **a** Typical changes in SaO_2_ images of the lower lobes, and the correlation between SaO_2_ calculated from SaO_2_ imaging at an FiO_2_ of 1.0 and the P/F ratio in the lower pulmonary vein, measured by blood-gas analysis. **b** Comparison of SaO_2_ calculated from SaO_2_ imaging in the top, middle, and bottom regions of the lower lobes between suitable and non-suitable cases

Figure 4a shows typical changes in SaO_2_ images of the lower lobes during the recondition and evaluation phases at 1 h in suitable and non-suitable cases. When FiO_2_ was changed in the evaluation phase, SaO_2_ in suitable cases remained close to 100% across most regions of the lower lobes (red area), whereas SaO_2_ was markedly lower (green or blue areas) in non-suitable cases. A strong positive correlation was observed between lower lobe SaO_2,_ calculated from SaO_2_ imaging at an FiO₂ of 1.0, and the P/F ratio in the lower pulmonary vein, measured by blood-gas analysis (*R* = 0.855, *P* < 0.01). Figure 4b compares SaO_2_ calculated from SaO_2_ imaging at an FiO_2_ of 1.0 in the top, middle, and bottom regions of the lower lung lobes between suitable and non-suitable cases. In all regions, SaO_2_ in non-suitable cases was significantly lower than that in suitable cases (*P* < 0.01).

These findings indicate that optical SaO_2_ imaging of the lower lobes during EVLP can serve as a quantitative and non-invasive method for assessing pulmonary oxygenation.

## Real-time lung weight measurement as an early indicator of transplant suitability [15]

**Fig. 5:**
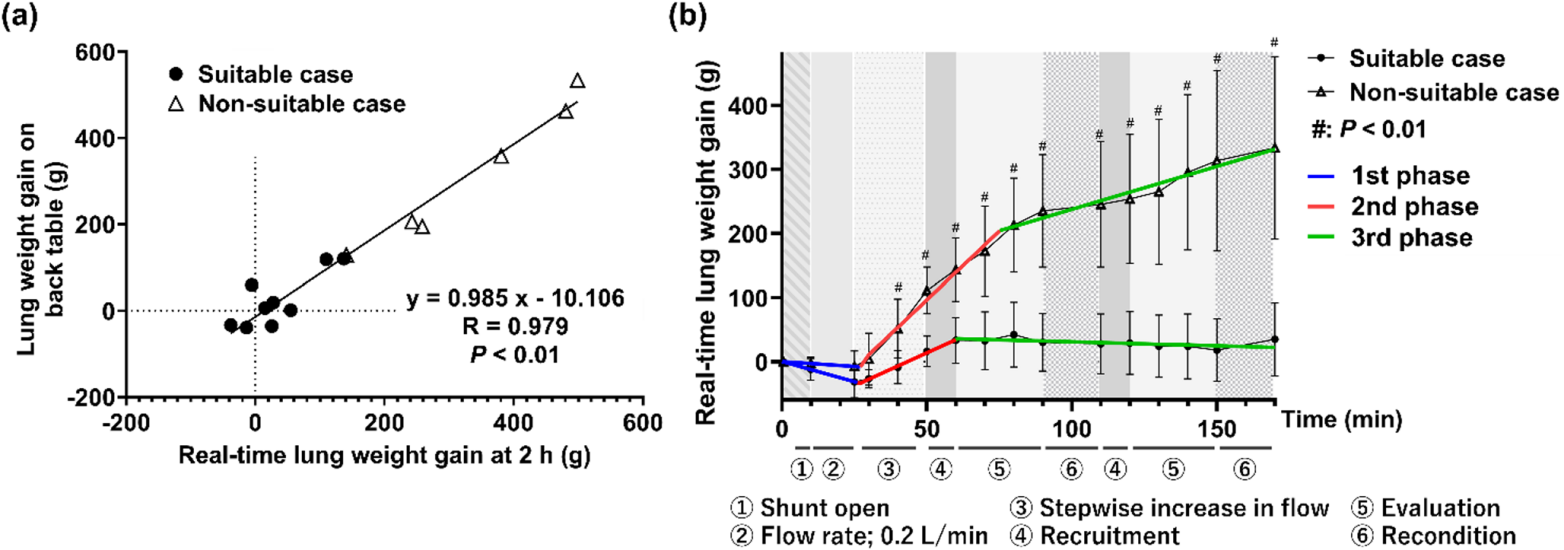
Real-time lung weight measurement for the assessment of pulmonary function. **a** Comparison between real-time lung weight gain after 2 h of EVLP and lung weight gain measured on the back table. **b** Trends in real-time lung weight gain in suitable and non-suitable cases during 2 h of EVLP

Real-time lung weight measurement was performed as an early indicator of transplant suitability during EVLP. For this, two load cells (Load cell Sensor 0–5 kg, Uxcell Co. Ltd., Hong Kong) were installed under the organ chamber. To minimize measurement artifacts unrelated to lung weight changes, a “non-touch period” was applied, which was defined as the interval when the lungs and organ chamber were not touched, and perfusion parameters remained stable. Lung weight gain was continuously measured during each non-touch period, and a constant rate of change was assumed until the next non-touch period. By repeating this procedure, continuous lung weight gain throughout the entire EVLP period was obtained. To validate this method, the real-time lung weight gain during 2 h of EVLP was compared with the difference in back-table lung weight gain measured before and after EVLP. A strong positive correlation was observed between the two measurements (*R* = 0.979, *P* < 0.01; Fig. 5a).

Figure 5b compares the trends in real-time lung weight gain in suitable and non-suitable cases. Lung weight gain in non-suitable cases was significantly higher than that in suitable cases after 40 min of perfusion (51.6 ± 46.0 g vs. −8.8 ± 25.7 g; cut-off = + 12 g, AUC = 0.907, *P* < 0.01). In breakpoint analysis using piecewise linear regression, the trend in real-time lung weight gain was divided into three phases. In the first phase (blue line), representing the low-flow period, lung weight gain was lower in both cases. This may have occurred because the osmotic pressure of the perfusate was lower than that of lung tissue, resulting in fluid movement from the tissue to the perfusate. In the second phase (red line), lung weight gain increased in both cases, particularly in non-suitable cases. As the flow rate was increased to 100% of the estimated cardiac output, the perfusate was distributed throughout the entire lungs, resulting in significant fluid leakage. In the third phase (green line), lung weight gain remained stable in suitable cases, indicating the absence of edema progression. In contrast, it continued to increase slightly in non-suitable cases, reflecting the accumulation of extracellular fluid in the interstitial space during the second phase, which suppressed further leakage.

These results indicate that real-time lung weight measurement during EVLP has the potential to enable early assessment of transplant suitability.

## Conclusions

In this study, we developed three non-invasive imaging and monitoring techniques for evaluating pulmonary function during EVLP: (1) lung thermography during the initial reperfusion period to assess pulmonary function, (2) optical SaO_2_ imaging for the assessment of pulmonary oxygenation, and (3) real-time lung weight measurement as an early indicator of transplant suitability. These methods proved effective for the quantitative and early assessment of pulmonary function during EVLP. Since these approaches enable non-invasive evaluation, they are expected to be applicable in clinical EVLP in the near future.
